# Self-organized chains of nanodots induced by an off-normal incident beam

**DOI:** 10.1186/1556-276X-6-432

**Published:** 2011-06-17

**Authors:** Seungjun Lee, Lumin Wang, Wei Lu

**Affiliations:** 1 Department of Mechanical Engineering, University of Michigan, Ann Arbor, MI 48109, USA; 2 Department of Materials Science and Engineering, University of Michigan, Ann Arbor, MI 48109, USA

## Abstract

We propose a model to show that under off-normal bombardment of an incident ion beam, a solid surface may spontaneously form nanoscale dots lining up into chains perpendicular to the incident beam direction. These dots demonstrate a highly ordered hexagonal pattern. We attribute the self-organization behavior to surface instability under concurrent surface kinetics and to a shadow effect that causes the self-alignment of dots. The fundamental mechanism may be applicable to diverse systems, suggesting an effective approach for nanofabrication.

## Introduction

Self-organized nanostructures have wide applications from functional materials to advanced electronic and optical devices [1,2]. Recent experiments demonstrated ion beam sputtering as a promising approach to generate various self-organized nanostructure patterns over a large area [3-8]. In this process, surface materials on the target are sputtered away by incoming ions, and the interplay between sputter-induced roughening and surface smoothening produces patterns such as ripples and dots. The feature size and morphology of these patterns are affected by parameters such as the incident ion beam flux, the beam energy, and the material of the substrate. Among them, the incident angle of the ion beam is an important factor to select the formation of different patterns. Normal bombardment produces hexagonally ordered dots [7], while off-normal bombardment produces ripples [4]. However, by rotating a sample simultaneously during off-normal sputtering, ordered dots can be obtained [3]. It was generally believed that sample rotation is necessary during off-normal bombardment to produce isotropic sputtering so that a pattern of dots can form.

Recently, the experiment of off-normal bombardment of Ga ion beam on a GaAs substrate showed an intriguing finding [9]. Hexagonally ordered dots were obtained even without sample rotation. More interestingly, the dots formed chains aligned perpendicular to the incident beam direction. A unique feature of this experiment is preferential sputtering, which refers to higher sputtering yield of certain element in the target and therefore causes a deviation of its surface composition from the original state [10]. For a GaAs substrate, the two elements (Ga and As) have different sputtering yield. The element As is more likely to be sputtered away, leaving a surface layer composed mostly of Ga. These Ga atoms diffuse on the surface and nucleate to form dots. This intriguing behavior to form nanoscale features calls for a new understanding.

Several models have been suggested to account for the pattern formation by an incident beam [11-14]. Most are rooted in the theory of Bradley and Harper [15], where the local sputtering rate depends on the surface curvature and the incident angle of the beam, leading to surface instability. However, the model cannot explain phenomena such as the saturation of the ripple amplitude and kinetic roughening. To account for these effects, the model was extended to include nonlinearity. For example, a nonlinear term, ∇^2^*h*, was introduced, where *h *is the surface height. This term leads to a finite saturated surface ripple amplitude after a long time of evolution [16]. To account for kinetic roughening, the model was further improved by adding a conserved KPZ term, ∇^2^(∇*h*)^2^, a higher-order term in Sigmund's theory [17]. These models necessarily generate ripples under off-normal bombardment because of anisotropic sputtering. In contrast, no ripples were observed during the preferential sputtering of GaAs. In this paper, we propose a model and the simulation to describe the dynamics of ordered dot formation and the alignment behavior under an off-normal beam. The fundamental mechanism may be applicable to diverse systems, suggesting a potential novel approach for nanofabrication.

## Model

We represent the substrate surface with a spatially continuous and time-dependent function, *h*(*x*,*y*,*t*), where *x *and *y *are axes parallel to the substrate surface and *t *is time. Starting from an initially flat surface, the formation of surface morphology and its evolution are captured by the change of *h *in the *z *direction. We consider concurrent surface kinetics including diffusion, redeposition, and sputtering. The time evolution of the surface is given by:(1)

The first term represents mass conservation, where **J **is the diffusion flux of Ga on the surface. The second term, *ρh*, accounts for the redeposition of sputtered atoms, which settle down on the surface again after traveling in the air [18]. The coefficient, *ρ*, describes the rate of redeposition. For a fixed coordinate, this term should be formulated as , where  is the spatial average of the surface height [18]. This term describes the phenomenon that atoms above the average height tend to be sputtered and redeposited on the surface below the average. Here, we use a moving coordinate such that the zero height coincides with the surface average and the  term is dropped. This paper focuses on surface morphology; thus, the average height change due to sputtering or redeposition is irrelevant. The third term,* β*(∇*h*)^2^, describes the tilt-dependent sputtering yield, which affects the saturation of growth [19]. The sputtering rate, *β*, is dependent on the beam flux and energy. Using a flat surface (∇*h *= 0) as a reference, the sputtering yield decreases with the slope. Thus, those regions with larger slopes tend to increase heights relative to the flat regions.

The diffusion flux, **J**, can cause either roughening or smoothening of the surface depending on the driving forces. We consider the net supply of Ga atoms on the surface for the roughening mechanism and the surface energy as well as the shadow effect for the smoothening mechanism. The roughening mechanism in ion beam bombardment is usually modeled by the theory of Bradley and Harper, which explains the surface instability by curvature-dependent energy dispersion, a process that happens by the removal of atoms similar as etching. In this case, the induced nanostructures such as ripples or islands are composed of the same material as that of the substrate. However, the dots shown in the GaAs experiment have different compositions from that of the substrate, suggesting that the diffusion of atoms plays an important role. In the experiment, Ga atoms are enriched on the surface due to preferential sputtering of As as well as the deposition of Ga from the ion beam. Enriched Ga atoms nucleate and grow into dots as they diffuse. The nanostructures formed by diffusion-driven roughening appear like droplets or bubbles [20,21]. They are amorphous and have a hemi-spherical shape rather than partially amorphous and form a cone shape [7,8] or ripples. They are usually observed at relatively high energy of ion beam over 10 keV, which is more likely to promote the preferential sputtering and high mobility of the diffusing atoms. Ripple structure induced by diffusion-driven roughening is hardly observed because highly mobile atoms tend to form droplets rather than longish ripples. The latter is usually generated by sputtering-driven roughening [22-25]. In this paper, we describe the growth of dots as an uphill mass flow along the slope, *α*∇*h*, where *α *is the growth rate that can be affected by the diffusing velocity of atoms and the sputtering yield. This term properly captures the instability and growth of dots due to the supply of atoms from the perimeter of the dot. This term is isotropic because atoms are supplied from all direction. Because it is not related to the angle of the incident beam, ripples do not appear in our model at off-normal bombardment, which is consistent with experimental observations.

The smoothening effect due to surface energy is considered in the following way. The chemical potential of atoms on the surface can be expressed by *μ *= *Kγ*Ω [26], where *K *is the surface curvature, *γ *is the surface energy per unit area, and Ω is the atomic volume. The curvature can be expressed by the second derivative of the surface height *K *= -∇^2^*h*. The atoms on the surface tend to move to regions with lower chemical potential, giving a diffusion flux of -*D*_*T*_∇*μ*, where *D*_*T *_is diffusion coefficient. Denote *λ *= *D*_*T*_*γ*Ω, we get a diffusion flux of *λ*∇(∇^2^*h*).

Next, we consider the shadow effect. In the shadow zone, where the ion beam is blocked by the dots during off-normal bombardment, the sputtering is weakened. The stronger sputtering on the top of dots (∇^2^*h *< 0) drives mass diffusion towards the shadowed valleys (∇^2^*h *< 0). The diffusing direction follows the gradient, ∇(∇^2^*h*). We represent this shadow effect by an additional surface smoothening term, which is similar to the surface energy term but modified in two aspects. Firstly, the shadow effect happens only along the direction of the incident beam. Without losing generality, we assume that the beam is within the *x*-*z *plane. Then, the shadow effect only happens along the *x *direction. Secondly, a surface higher gets more sputtering and deeper in the valley gets less sputtering. To the first order approximation, we assume that the smoothening flux is proportional to *h*. Following the form of surface energy, the corresponding mass flux can be written as *η*{**i ***h*∇(∇^2^*h*)}**i**, where **i **is the unit vector in the *x *direction and *η *is the coefficient. Note that the *h *before the gradient operator makes this term nonlinear, which becomes important only after the surface has developed sufficient roughness. Otherwise, this term would affect the early stage of simulations, whose anisotropic smoothening effect would generate ripples not observed in experiments. The magnitude of *η *will depend on the incident angle, *θ*, between the incident beam and the *z *axis.

Consideration of all the contributions gives the following diffusion flux:(2)

**Figure 1:**
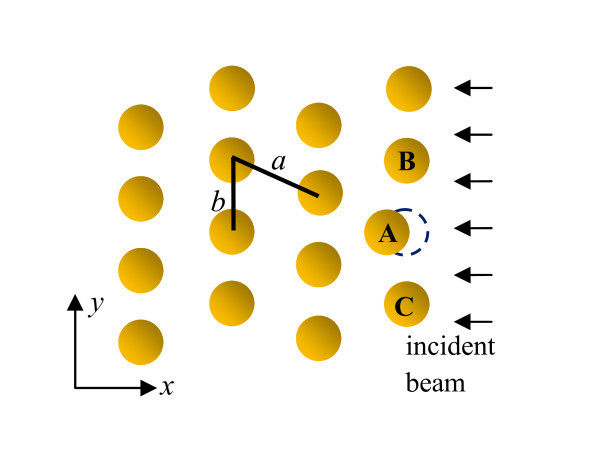
**Schematic of a hexagonal pattern of dots lined up along the *y *axis**. The formed line is perpendicular to the direction of the incident beam. Dot A would be partially shadowed by B and C if it shifts to the left, when mass accumulation at its front would bring it back to line up with B and C. Anisotropic smoothing causes the distance between dots anisotropic, i.e., *a > b*.

Now, we discuss how the shadow effect causes the dots to line up into chains. Consider a hexagonal pattern of dots as shown in Figure 1. These dots line up into chains along the *y *axis. Dot A would be partially shadowed by B and C if it shifts to the left, when mass accumulation at its front would bring it back to line up with B and C. Similarly, dot A would be exposed to more sputtering if it shifts to the right and would gradually move back to be in-line with B and C. The anisotropic smoothing given by the third term in Equation 2 causes the wavelength in the *x *direction to be larger than that in the *y *direction. As a result, the distance between dots is not isotropic, i.e., *a > b *in Figure 1. This behavior is consistent with experimental observations.

To facilitate numerical simulations, Equations 1 and 2 can be expressed into dimensionless forms with *h*, *x*, and *y *normalized by a length scale *l*_0 _and *t *normalized by a time scale, *t*_0_. Then, parameters *ρ*, *β*, *α*, *λ*, and *η *are normalized by 1/*t*_0_, *l*_0_/*t*_0_, *l*_0_^2^/*t*_0_, *l*^4^_0_/*t*_0_, and *l*^3^_0_/*t*_0_, respectively. The dimensionless equations appear the same as Equations 1 and 2, except that the symbols now represent the corresponding normalized values, such as *h *represents *h*/*l*_0_. Below, we always refer to the normalized quantities.

## Results and discussion

The finite difference method was used to solve Equation 1 in its dimensionless form. The calculation domain size was taken to be 200 × 200. Periodic boundary conditions were applied. The grid spacing and time step were taken to be Δ*x *= Δ*y *= 0.5 and Δ*t *= 0.01, which correspond to a physical spacing of 6 nm and a physical time step of 1.8 ms. The initial surface morphology was constructed by adding to a flat surface a small random perturbation with magnitudes between 0 and 10^-5^.

**Figure 2:**
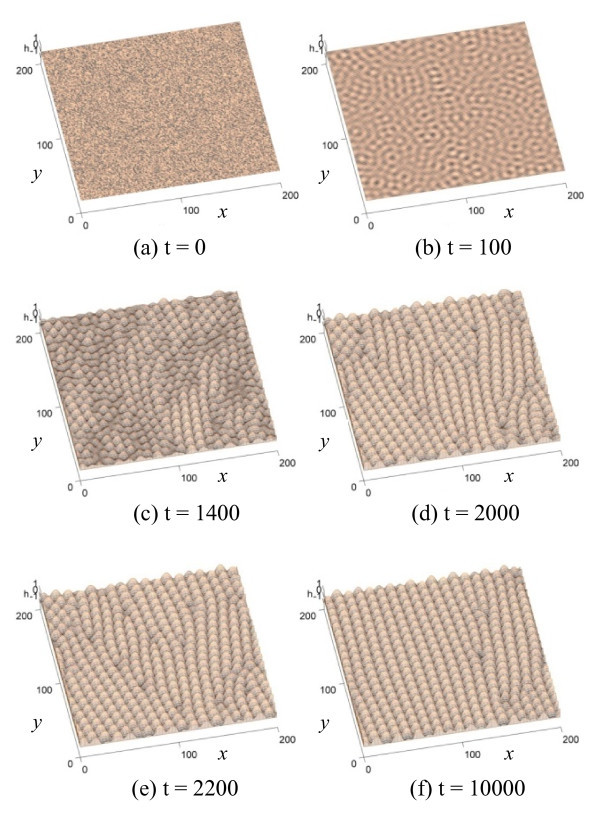
**An evolution sequence showing that self-organized dots emerge, line up, and form chains**.

**Figure 3:**
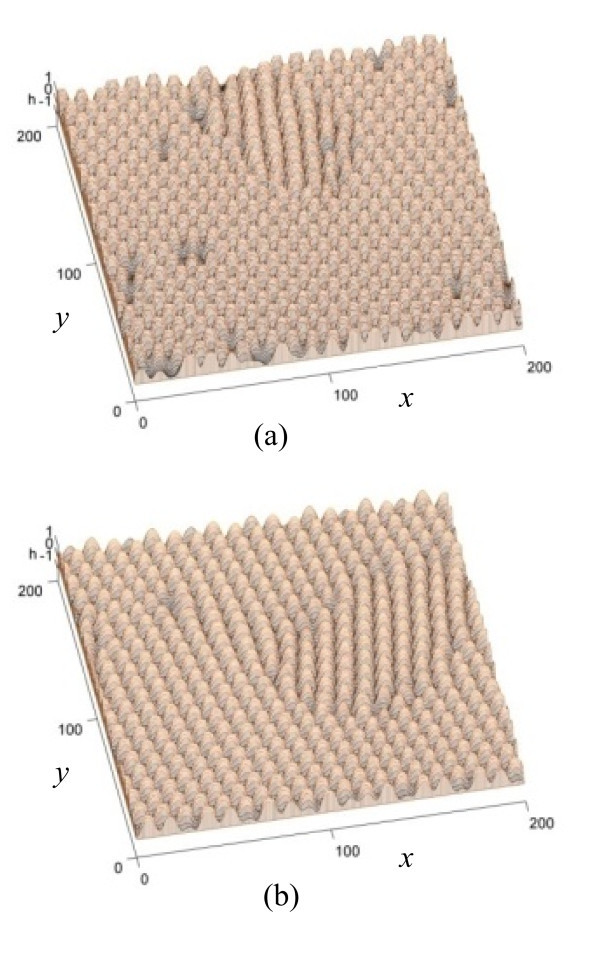
**Simulation results at *t *= 10,000 for different values of *η***. The results reveal how the strength of the shadow effect affects the pattern. (**a**) No shadow effect (*η *= 0) and (**b**) weak shadow effect (*η *= 0.5).

Representative simulation results are shown in Figures 2 and 3. The following normalized parameters were chosen: *ρ *= 0.24, *β *= 1, *α *= 1, and *λ *= 1 [27]. Figure 2 shows an evolution sequence for *η *= 1.0 from *t *= 0 to *t *= 10,000. Figure 2a shows the initial substrate surface at *t *= 0. After a short time of bombardment, small peaks quickly emerge and form a wavy chain pattern, as shown in Figure 2b. Linear terms are dominant during the early stage of evolution. The nonlinear term representing the shadow effect does not reflect itself significantly in the result. Dots start to emerge and grow quickly after *t *= 1,000, as shown in Figure 2c for *t *= 1,400. As of now, the dots are randomly distributed without showing any particular order. The height growth of dots slows down after *t *= 2,000, since the nonlinear term starts to affect the growth. Figures 2d and 2e show that the dots start to line up and form short chains. Overall, these short chains appear to orientate along the *y *axis, though the orientation of a single chain is less definite. During this stage, the dominating behavior is the change of the location of dots particularly at dislocation regions, while their heights remain almost constant. Over time, the chains become more ordered. Figure 2f shows that at *t *= 10,000, the chains are clearly aligned along the *y *axis, which is perpendicular to the incident beam direction. The dots form a hexagonal pattern and their sizes are uniform. These simulation results are consistent with experimental observations [9].

Figure 3 shows simulation results at *t *= 10,000 for different values of *η*, revealing how the strength of the shadow effect affects the pattern. The parameter *η *is a function of the incident angle, where *η *= 0 corresponds to normal bombardment, or zero incident angle between the incident beam and the *z *axis. The magnitude of *η *increases with the incident angle. Figure 3a shows that no chain is formed when there is no shadow effect or *η *= 0. The dots simply form a hexagonal pattern. Figure 3b shows that with *η *= 0.5, chains appear to form but are not perfectly aligned. The comparison with Figure 2f clearly shows that stronger shadow effect leads to well-aligned chains perpendicular to the beam direction.

## Conclusions

Our model and simulations have revealed how self-organized dots emerge, line up, and form chains during ion beam sputtering. These simulations show the importance of the shadow effect, which happens only during off-normal bombardment and leads to chains perpendicular to the incident beam direction. In addition, it is shown that the chains of dots are not formed by an initial ripple generation along *y *followed by a subsequent process to break up these ripples into dots. Instead, the dots emerge at the early state of evolution and then gradually rearrange to form chains. These results are consistent with experiments. The study in this paper will provide insight into the self-organization process and provide guidance to extend the approach for nanofabrication. For instance, similar mechanism may be applied to other compound systems as a general approach to form ordered nanodot patterns. Our study suggests that high mobility is essential, which gives a hint that it may be necessary to raise the temperature close to the melting point to initiate the mechanism.

## Competing interests

The authors declare that they have no competing interests.

## Authors' contributions

SL carried out the modeling and numerical simulation and drafted the manuscript. LMW provided experimental observations. WL guided the modeling and helped to draft the manuscript. All authors read and approved the final manuscript.
